# Clinical and radiographic assessment of composite CAD/CAM endocrowns and stainless steel crowns for endodontically treated first permanent molars in Egyptian children: randomized controlled pilot study

**DOI:** 10.1186/s12903-025-06192-y

**Published:** 2025-05-31

**Authors:** Basheer Ali Mabkhot, Sheriene Ezz Eldin Taha, Shaimaa Mohamed Sabry

**Affiliations:** 1 Researcher of Pediatric Dentistry and Dental Public Health, Faculty of Dentistry, Cairo University, Cairo, Egypt; 2 Professor of Pediatric Dentistry and Dental Public Health, Faculty of Dentistry, Cairo University, Cairo, Egypt; 3 Associate Professor of Pediatric Dentistry and Dental Public Health, Faculty of Dentistry, Cairo University, Cairo, Egypt

**Keywords:** Pediatric Dentistry, Child, First Permanent Molar, Molar, Stainless Steel, Composite CAD/CAM Endocrown, Crowns, Computer-Aided Design, Pilot Projects, Egypt

## Abstract

**Background:**

Dental caries in first permanent molars (FPMs) constitutes a worldwide health concern. Managing FPMs with deep dental caries in children poses a significant struggle for dental practitioners. The objective of this research is the clinical and radiographic evaluation of composite CAD/CAM endocrowns as restoration versus stainless steel crowns (SSCs) for endodontically treated FPMs in children.

**Patients and methods:**

This pilot study evaluated 24 children with deep caries in FPMs attending the dental clinic of Pediatric Dentistry and Dental Public Health Department, Faculty of Dentistry, Cairo University, Egypt. They were randomly assigned to receive either a composite CAD/CAM endocrown (Group I) or an SSC (Group II). Clinical evaluations (postoperative pain, crown retention, patient/parent satisfaction) were performed at one week, three, six, nine, and 12 months. Radiographic assessments (tooth fracture) were done at one week, six months, and 12 months.

**Results:**

Prior to the intervention, no statistically significant differences were observed between the two groups. No significant differences in postoperative pain scores were observed between groups. Both groups showed good crown retention, with one clinical failure in each group. Patient satisfaction was higher for endocrowns compared to SSCs at three, six, nine, and 12 months. No root fractures were observed in either group.

**Conclusion:**

Both composite CAD/CAM endocrowns and SSCs showed similar clinical and radiographic outcomes, except for patient satisfaction. Clinicians may consider composite CAD/CAM endocrowns if patient satisfaction is the primary concern.

**Trial registration:**

Current Controlled Trial NCT05250609. Registered on 22/02/2022, retrospectively registered. https://clinicaltrials.gov/study/NCT05250609.

## Introduction

Dental caries is a globally common condition that impacts individuals across all age groups, ranging from children to older adults [1]. The first permanent molars (FPMs) are the first teeth that emerge in the mixed dentition [2]. The mixed dentition stage often coincides with a decline in oral hygiene practices due to various factors [3]. Children at this age may be less concerned with oral care and may experience emotional stress. Also, a diet rich in sugary foods, coupled with the natural process of tooth replacement, can increase the risk of dental caries, especially in the FPMs [3]. Parents often lack awareness of FPMs, especially since they erupt without the shedding of primary teeth, which can further compromise their health [4].

Dental caries in FPMs is a global concern. Studies from various regions, including India, China, Saudi Arabia, Romania, and Sudan, have reported varying prevalence rates [5–8]. In general, this prevalence tends to increase with age, with higher rates observed in younger age groups. Mandibular FPMs, with their complex morphology and earlier eruption, are more susceptible to caries than maxillary FPMs [7, 9]. FPMs play an important role in establishing the occlusion (key of occlusion) and creating space for the eruption of other permanent teeth. If this tooth is lost early, it may lead to disruptions in the eruption and positioning of other permanent teeth [5]. They significantly contribute to the growth and function of the stomatognathic system [10].

Stainless steel crowns (SSCs) are commonly applied to treat primary molars after extensive caries removal and pulp treatment [11]. While they are not a permanent solution for permanent teeth, they can be used as interim restorations until definitive prosthetic treatment [12–15]. SSCs are cost-effective for treating FPMs, offering durability and full tooth coverage. They are easy to place and require minimal technique sensitivity. However, their limited aesthetic appeal may be a concern for some patients [12, 16]. Additionally, SSCs might negatively impact periodontal health [15, 17].

Preformed zirconia crowns (PZCs) are often chosen to restore endodontically treated FPMs due to their aesthetic appeal. However, the clinical time necessary for preparing and placing PZCs is substantially greater, approximately double that of SSCs [18]. Direct composite restorations, which are considered another aesthetic option for restoring FPMs, demonstrate a high incidence of failure characterized by marginal leakage, discoloration, and fracture [19].

Endocrowns are a conservative restoration choice for teeth exhibiting considerable coronal caries (up to 50%). They are particularly useful when there is limited interocclusal space or inadequate clinical crown length [20]. Endocrowns can also be a good alternative to intra-radicular posts, especially in cases of short, curved, calcified, narrow roots or root canal instrument fractures [20]. Various materials, including composite CAD/CAM, have been used to fabricate endocrowns [21]. Composite CAD/CAM endocrowns were selected for this study due to their specific advantages, such as elasticity similar to dentin, better marginal stability, and less abrasive properties compared to ceramic restorations. These characteristics allow for more even stress distribution and the potential for in-mouth adjustments and repairs [22].

Although the limitations of SSCs, PZCs, and direct composite restorations are well-documented, comparative research assessing the clinical and radiographic performance of composite CAD/CAM endocrowns as an alternative for pediatric patients with endodontically treated FPMs is scarce. This study aims to clinically and radiographically assess composite CAD/CAM endocrowns as interim restorations compared to SSCs for endodontically treated first permanent molars (FPMs) in children. The null hypothesis of this study is that there is no significant difference in the clinical and radiographic outcomes between endocrowns and SSCs in pediatric patients.

## Patients and methods

### Research design

A pilot study with a randomized controlled design and an equal 1:1 allocation was carried out at the Department of Pediatric Dentistry and Dental Public Health, Faculty of Dentistry, at Cairo University, in Egypt.

### Ethical approval

This study adhered to ethical standards sanctioned by the Research Ethics Committee of Cairo University’s Faculty of Dentistry (approval reference: [3, 4, 22]), with all dental procedures conducted accordingly. Consent in written form was secured from the parent or guardian of each participating child. Furthermore, registration of the research protocol was completed on ClinicalTrials.gov with the identifier NCT05250609 on 22/02/2022.

### Sample size calculation

This pilot study included 24 restorations (12 in each group). Due to the absence of prior research or pilot data specific to this intervention, a precise estimation of the effect size is not feasible. Therefore, an initial sample size of 20 cases (10 per group) was estimated based on expert opinion. To compensate for possible participant dropout during the follow-up period, the sample size was expanded to 24 participants, with 12 assigned to each group.

### Study design and participant recruitment

This research utilized a prospective, randomized, controlled approach, dividing participants into two evenly balanced groups. Participants were recruited from the Pediatric Dentistry and Dental Public Health Department outpatient clinic at Cairo University. Following the acquisition of explicit permission from parents or legal guardians, demographic, medical, and dental information was collected.

### Randomization and group allocation

Twenty-four participants were evenly divided into two groups through random assignment: an intervention group and a control group. The intervention group received composite CAD/CAM endocrowns, whereas the control group received SSCs.

### Eligibility criteria

#### Inclusion criteria

- Children aged 10–13 years who were cooperative during treatment.
- Parental or guardian consent to participate in the study.
- Mandibular FPMs with irreversible pulpitis or necrotic pulp requiring root canal treatment with a closed apex.
- Teeth with at least two to three sound walls.
- Absence of internal or external root resorption.
- Normal occlusion without parafunctional habits.

#### Exclusion criteria

- Excessive patient movement or difficulty in cooperation.
- Underlying systemic diseases.
- Special healthcare needs.
- Poor oral hygiene.

### Participant allocation

**Fig. 1:**
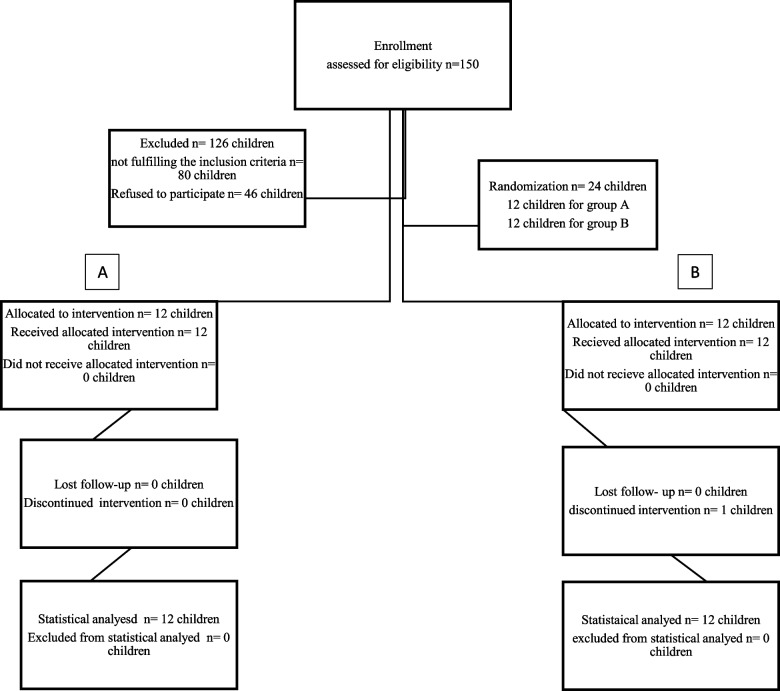
Flow chart of the trial enrollment, randomization, allocation, follow-up, and analysis according to CONSORT guidelines

This study adhered to CONSORT guidelines for transparent reporting. To ensure unbiased group assignment, a computer program (https://www.randomizer.org/) was used to generate a random sequence of participant allocations. The supervisor oversaw this process. The participants were subsequently split into two cohorts (Group A and Group B) according to their random assignment numbers. This randomization process guaranteed that the type of restoration (preparation for a composite CAD/CAM endocrown in Group A or preparation for SSCs in Group B) was determined solely by chance (Fig. 1). Every participant was given a sealed, non-transparent envelope that held their designated number, guaranteeing that the allocation concealment until the initiation of the treatment procedure.

### Blinding

To minimize bias, the statistician remained unaware of patient group assignments throughout the study. However, due to the nature of the clinical procedures, the principal investigator could not be blinded. To enhance objectivity, two independent experts assessed outcomes on two separate occasions, spaced five days apart, to ensure intra- and inter-rater reliability.

### Clinical steps

Clinical examinations and treatments were conducted using a dental unit (Knight by Midmark, USA). Digital radiographs were obtained with a phosphor plate machine (KaVo Scan eXam™, Dental GmbH, Germany) to evaluate pre- and post-treatment conditions. Endodontic procedures were carried out using an apex locator (WoodpexIII Plus-L-U, Woodpecker, China) and a rotary endo motor (E-com + Cordless Endo Motor, Woodpecker, China) with M-Pro rotary files.

The control group required two visits, while the intervention group needed three. A two-week interval was maintained between the first and second visits for pain assessment. For the intervention group, an additional follow-up visit was scheduled 24–48 h after the second appointment.

### The first visit for both groups

Root canal treatment (RCT) was carried out on the FPM, following the protocol for the management of irreversible pulpitis and necrotic pulp [23]. A post-treatment X-ray was conducted to confirm the root canal procedure’s success, after which a temporary restoration was applied.

### The second visit for the control groups

A Tofflemire matrix band (Medicem, Germany) was used to fill the tooth with Glass Ionomer Cement (Medicem, Germany) after Root Canal Treatment. SSCs (3M™ Stainless-steel for permanent Molar Crowns, US) of appropriate size were selected, considering the mesio-distal width of the tooth.

For tooth preparation, approximately 1.5–2 mm of occlusal reduction was performed, followed by mesial and distal reductions. Conservative preparation techniques were employed to maintain tooth structure, with a smooth feather-edge finishing line placed just below the gingival margin and rounded line angles. The crowns were cemented using Glass Ionomer cement (Medicem Glass ionomer luting cement, GmbH, Germany) [24].

### The second visit for the intervention groups

**Fig. 2:**
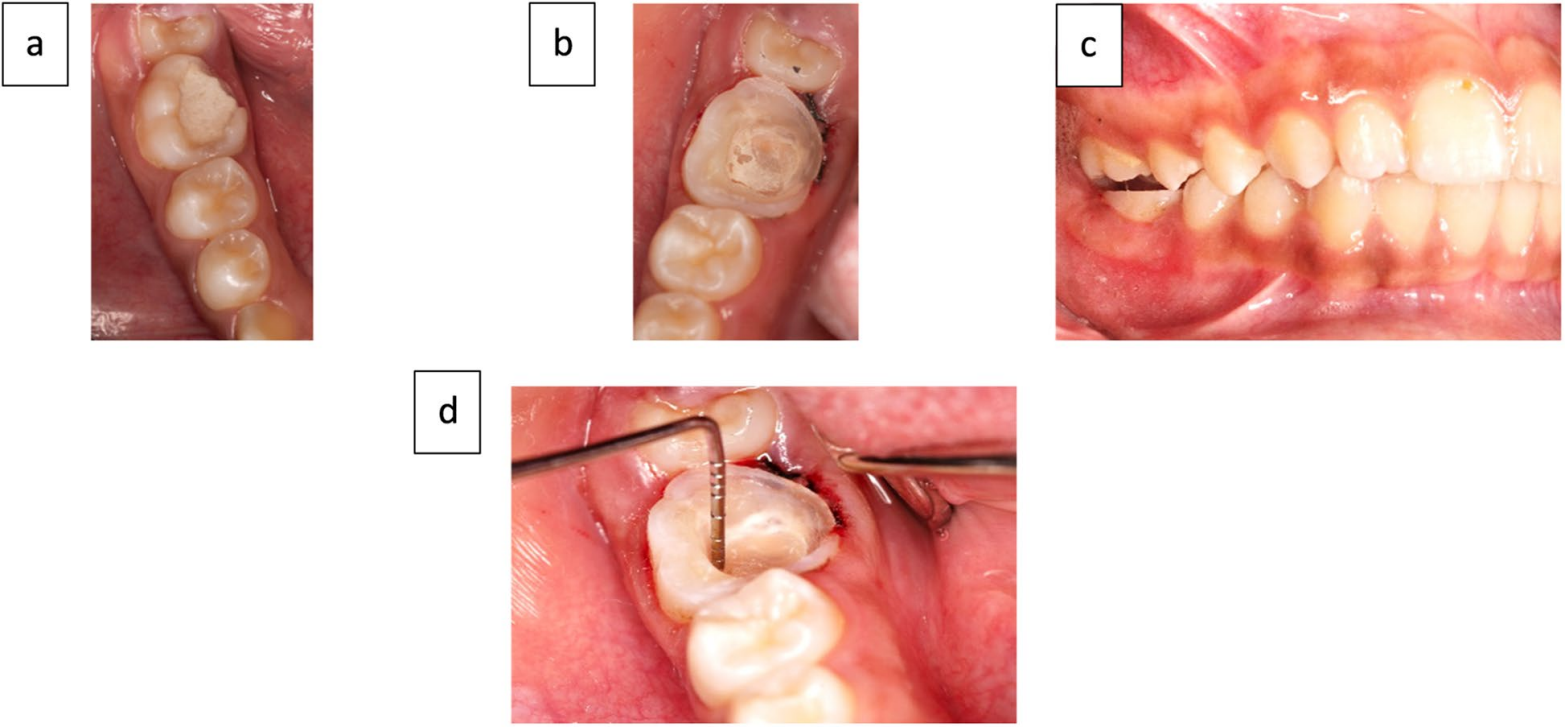
(**a**) PFM after RCT, (**b**) Applied flowable composite, (**c**) 1.5 mm occlusal clearance, (**d**) 3 mm depth of pulp chamber

The tooth was anesthetized and isolated with a rubber dam, and the temporary filling was removed using a fissure diamond bur. The pulp chamber was cleaned and etched with 37% phosphoric acid gel (Meta Biomed, Republic of Korea), followed by bonding with All-Bond Universal adhesive (BISCO, USA). The orifices of the pulp chamber were sealed with a flowable composite resin (Nano Hybrid Flowable Composite Resin, Meta Biomed, Republic of Korea) (Fig. 2a, b) [25, 26].

To prepare the tooth, a tapered stone was used to create depth grooves for adequate occlusal reduction. A wheel stone was then employed to establish a 1.5 mm clearance on the occlusal surface. The occlusal surface was flattened to form a butt joint, and the junction between the occlusal and mesial cavity was rounded to create a cervical shoulder (Fig. 2c) [25, 26].

To create the desired divergence of the inner cavity walls, a tapered stone bur with a rounded tip (TF-21, WR-13, AZDENT Diamond Bur Wheel, Taper Round End Cone, China) was utilized. A gingival retraction was achieved by placing a retraction cord prior to impression taking. The depth of the pulp chamber and the width of the prepared cavity walls were measured using a periodontal probe (Perio Prob, Dentart Instrum MFG. Co., Sialkot—Pakistan) to ensure a minimum depth of 3mm (Fig. 2d) [27].

A condensation silicone impression material (Zetaplus 900 ml, Oranwash L 140 ml, Indurent Gel 60 ml, Zhermack Zetaplus C-silicone Kit, Italy) was used to capture the prepared tooth structure. Additionally, a vinyl polysiloxane (VPS)-based bite registration material (O-Bite, DMG, Germany) was used to record the occlusal relationship between the maxillary and mandibular teeth. The mixed dental stone was poured into the impressions to create dental stone models. These models were then used for the fabrication of the composite CAD/CAM endocrown.

**Fig. 3:**
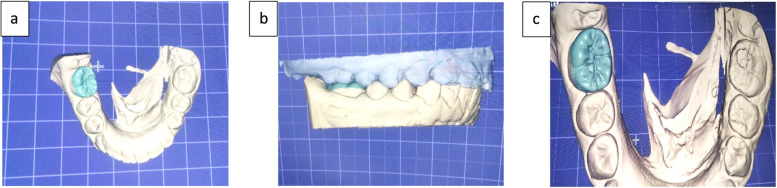
(**a**, **b**, **c) **Exocad program designing the endocrown

In the laboratory, a high-impact polymer composite block (BreCAM, Bredent GmbH & Co.KG, Germany) was utilized to fabricate the endocrown using a computer-aided design/computer-aided manufacturing (CAD/CAM) process. Initially, a Medit T710 scanner (Universadent Inc, Canada) captured digital impressions of the stone models (Fig. 3a, b, c). This data was then transferred to a dedicated software program compatible with the Bristol CAD/CAM system, specifically a Roland DWX-52D milling machine (Japan). Notably, all endocrown restorations were fabricated using this particular machine [27].

### Third visit: endocrown cementation

**Fig. 4:**
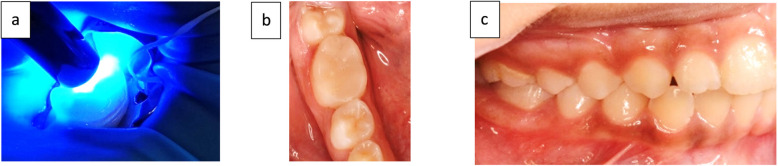
(**a**) Light curing the fitted fabricated endocrown on prepared FPM, (**b**, **c**) After cementation

Rubber dam isolation was implemented following local anesthesia administration. The tooth was etched for 40 s, rinsed, and dried before applying a bonding agent and light-curing for 40 s. The prefabricated composite CAD/CAM endocrown was etched and bonded extraorally. The endocrown was then cemented in place using a self-adhesive dual-cure resin cement (Nova Resin Cement, IMICRYL, Turkey). The cement was cured for 40 s with an LED light curing unit from multiple angles to ensure complete polymerization (Fig. 4a).

Excess resin cement was removed using a composite finishing bur (TC-21EF, TR-26F, AZDENT Finishing Short Thin bur, Taper Round bur, China). Dental floss was used to confirm the removal of excess cement from the mesial and distal proximal areas of the restoration (Fig. 4b, c). All procedures were standardized by having a single operator perform all steps [27].

### Outcome assessment

The study assessed outcomes pre- and post-operatively at 1 week and 3, 6, 9, and 12 months.

#### Postoperative pain

Postoperative pain levels were assessed with an adapted version of the Wong-Baker Faces Pain Rating Scale, ranging from 0 to 3. A rating of 0 denoted the absence of pain, 1 indicated slight discomfort, 2 signified moderate discomfort, and 3 corresponded to intense pain. The principal investigator directly questioned the patient about their pain experience [28].

#### Retention

The principal investigator assessed participants clinically and recorded the results as a binary outcome (Yes or No) [16].

#### Patient satisfaction

Satisfaction levels among patients and guardians regarding restorative treatment were evaluated using a modified 4-point scale (1 = very dissatisfied, 2 = dissatisfied, 3 = satisfied, 4 = very satisfied) [29]. To reduce subjectivity and potential bias, we defined criteria based on three parameters—pain, chewing ability, and aesthetic outcome—as follows:

1. Very Dissatisfied: Persistent pain, difficulty chewing most foods, poor aesthetic outcome (noticeable mismatch).
2. Dissatisfied: No persistent pain, mild chewing difficulty, fair aesthetic outcome (slight mismatch).
3. Satisfied: No persistent pain, comfortable chewing, good aesthetic outcome (minor, non-noticeable imperfections).
4. Very Satisfied: No persistent pain, unrestricted chewing, excellent aesthetic outcome (seamless integration).

#### Radiographic evaluation

Radiographic assessments were conducted by the assistant supervisor and radiographic supervisor at baseline, six months, and one year post-treatment. Root fractures were evaluated and categorized as either present or absent [30].

### Statistical analysis

Normality of numerical variables was evaluated through visual inspection of data distributions and application of Kolmogorov–Smirnov and Shapiro–Wilk tests. Age was found to be normally distributed, whereas all other numerical variables were non-normally distributed. Numerical data is presented as means with standard deviations or medians along with ranges, as applicable. Categorical data is summarized using frequencies and percentages. Comparisons between groups for categorical data were conducted using the Chi-square and Fisher's Exact tests.

A Student's t-test was used to evaluate the differences in mean age between the two groups for parametric analysis data. For non-parametric comparisons between groups, the Mann–Whitney U test was utilized. Intra-group changes over time were analyzed using Friedman's test. Where Friedman's test revealed significant differences, Dunn's post-hoc test was conducted to determine specific pairwise comparisons between time points.

The intraclass correlation coefficient (ICC) was used to evaluate both intraobserver and interobserver reliability, particularly the Kappa statistic. Statistical significance was defined as P ≤ 0.05. All analyses were conducted using IBM SPSS Statistics for Windows, Version 23.0 (IBM Corp., Armonk, NY).

## Results

The prospectively registered protocol NCT05250609 was followed in all aspects of its conduct, and there were no deviations from the intended treatment methods.

### Demographic characteristics

No statistically significant difference was observed between the mean ages of the two groups. Similarly, there was no significant difference in gender distribution between the groups, as shown in Table 1.

**Table 1:** Comparison of baseline characteristics between the two groups, including means (SD), frequencies (n), percentages, and statistical significance determined by Student's t-tests and Chi-square tests

	Endocrown Group (*n* = 12)	SSC Group (*n* = 12)	*P*-value
Age (Years)			0.097
Mean (SD)	11 (0.95)	11.58 (0.67)	
Gender [n (%)]			0.682
Male	5 (41.7%)	6 (50%)	
Female	7 (58.3%)	6 (50%)	

^*^Significant at *P* ≤ 0.05

### Clinical evaluation

#### Pain assessment (VAS Score)

##### Comparison between groups

No statistically significant differences in pain scores were observed between the two groups at preoperative, one-week, three-month, six-month, nine-month, and 12-month follow-up periods (*P* > 0.05), as shown in Table 2.

**Table 2:** Descriptive statistics of pain (VAS) scores, and the statistical significance of inter-group differences (Mann–Whitney U test) and intra-group changes over time (Friedman's test)

Time	Endocrown Group (*n* = 12)		SSC Group (*n* = 12)		*P*-value	*Effect size (d)*
	Median (Range)	Mean (SD)	Median (Range)	Mean (SD)		
Preoperative	3 (2–3) ^A^	2.83 (0.39)	3 (3–3) ^A^	3 (0)	0.148	0.286
1 week	0 (0–1) ^B^	0.25 (0.45)	0 (0–1) ^B^	0.08 (0.29)	0.284	0.286
3 months	0 (0–0) ^B^	0 (0)	0 (0–3) ^B^	0.5 (1.17)	0.148	0.286
6 months	0 (0–0) ^B^	0 (0)	0 (0–0) ^B^	0 (0)	1	0
9 months	0 (0–0) ^B^	0 (0)	0 (0–0) ^B^	0 (0)	0.338	0.274
12 months	0 (0–1) ^B^	0.08 (0.29)	0 (0–2) ^B^	0.18 (0.6)	0.900	0.166
*P*-value	< 0.001*		< 0.001*			
Effect size (*w*)	0.802		0.759			

^*^Significant at *P* ≤ 0.05, Different superscripts in the same column indicate statistically significant change by time

##### Changes within each group

Both groups exhibited a statistically significant reduction in pain scores over time. The endocrown group demonstrated a larger effect size (*P*-value < 0.001, Effect size = 0.802) compared to the SSC group (*P*-value < 0.001, Effect size = 0.759). In both groups, pairwise comparisons between time periods revealed that pain scores significantly decreased after one week, followed by non-significant changes at subsequent time points, as shown in Table 2.

#### Retention

##### Comparison between groups

After one week, all restorations in both groups were retained. Consequently, no statistical comparison was performed. At three, six, nine, and twelve months, the retention rates between the two groups were not statistically different (*P* > 0.05). Effect sizes were calculated to be (*P*-value = 1, Effect size = 0.478), (*P*-value = 1, Effect size = 1), (*P*-value = 1, Effect size = 1), and (*P*-value = 1, Effect size = 1), respectively, as shown in Table 3.

**Table 3:** Comparative analysis of retention between the two groups using Fisher’s Exact test, and within-group changes using Friedman’s test, along with frequencies (n) and percentages (%)

Time	Endocrown Group (*n* = 12)		SSC Group (*n* = 12)		*P*-value	*Effect size (Odds Ratio)*
	n	%	n	%		
1 week	12	100	12	100	Not computed because the variable is constant	
3 months	12	100	11	91.7	1	0.478
6 months	11	91.7	11	91.7	1	1
9 months	11	91.7	11	91.7	1	1
12 months	11	91.7	11	91.7	1	1
*P*-value	0.406		0.406			
Effect size (*w*)	0.083		0.083			

^*^Significant at *P* ≤ 0.05

##### Changes within each group

No statistically significant difference in retention rates was observed over time for either group (*P*-value = 0.406, Effect size = 0.083), as shown in Table 3.

### Radiographic evaluation

#### Fracture

**Fig. 5:**
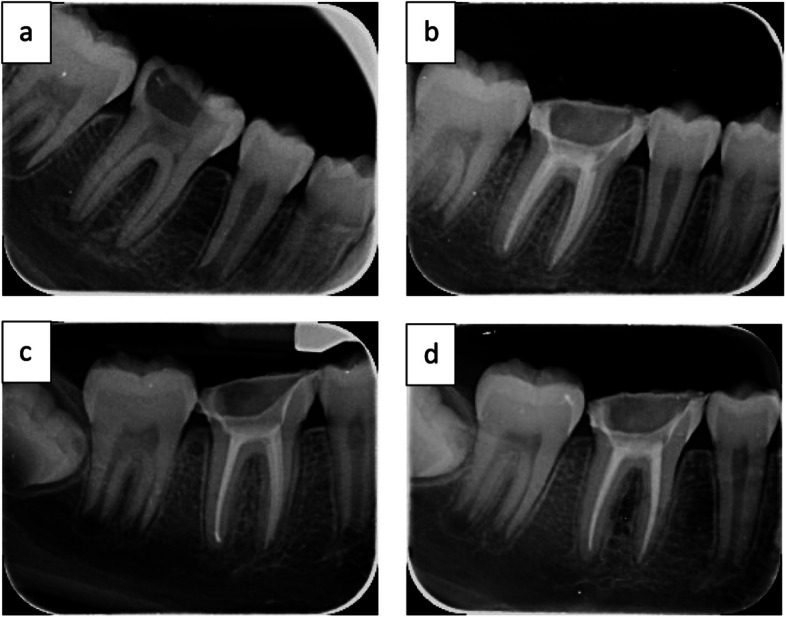
Radiographic films of the intervention group (**a**) preoperative, (**b**) postoperative, (**c**) postoperative 6 months, (**d**) postoperative 12 months

 All crowned teeth structures in the two groups had no root or bifurcation fractures, so no comparisons were performed (Fig. 5).

#### Patient satisfaction score

##### Comparison between groups

At the one-week follow-up, no statistically significant difference in patient satisfaction was observed between the two groups (*P* = 0.070, effect size = 0.434). However, at the three, six, nine, and twelve-month evaluations, patients in the endocrown group reported significantly higher levels of satisfaction compared to those in the SSC group (*P* = 0.014, effect size = 0.756; *P* = 0.025, effect size = 0.756; *P* = 0.045, effect size = 0.785; *P* = 0.045, effect size = 0.785, respectively) as shown in Table 4.

**Table 4:** Analysis of patient satisfaction scores, including descriptive statistics and the results of Mann–Whitney U tests for inter-group comparisons and Friedman's tests for intra-group changes

Time	Endocrown Group (*n* = 12)		SSC Group (*n* = 12)		*P*-value	*Effect size (d)*
	Median (Range)	Mean (SD)	Median (Range)	Mean (SD)		
1 week	4 (4–4)	4 (0)	4 (3–4)	3.75 (0.45)	0.070	0.434
3 months	4 (4–4)	4 (0)	4 (1–4)	3.42 (0.9)	0.014*	0.756
6 months	4 (4–4)	4 (0)	4 (2–4)	3.55 (0.69)	0.025*	0.756
9 months	4 (3–4)	3.92 (0.29)	4 (2–4)	3.45 (0.69)	0.045*	0.785
12 months	4 (3–4)	3.92 (0.29)	4 (2–4)	3.45 (0.69)	0.045*	0.785
*P*-value	0.406		0.160			
Effect size (*w*)	0.083		0.150			

^*^Significant at *P* ≤ 0.05

##### Changes within each group

Neither group demonstrated a statistically significant change in satisfaction scores over time (*P*-value = 0.406, Effect size = 0.083) and (*P*-value = 0.160, Effect size = 0.150), respectively, as shown in Table 4.

## Discussion

This research assessed both clinical and radiographic outcomes of composite CAD/CAM endocrowns compared to SSCs as interim restorations for endodontically treated FPMs in children over a one-year follow-up. The major findings reveal that both restoration types demonstrated comparable retention and radiographic outcomes with minimal failures. Postoperative pain decreased significantly within one week following RCT and remained stable thereafter in both groups. However, patient satisfaction was notably higher with composite CAD/CAM endocrowns, driven primarily by their aesthetic appeal. Based on these results, the null hypothesis is rejected—that there is no difference between the two restoration types—due to the significant difference in satisfaction despite similar clinical outcomes.

The clinical applicability of this study's outcomes depends on the consistent implementation of the intervention according to the study's methodology. The criteria of the present study were chosen because, at ages 10–13, mutilated FPMs are mature and indicated for RCT, providing an empty pulp chamber ideal for endocrown retention. Additionally, the presence of at least two sound tooth walls ensures adequate adhesion and stability. Excluding patients with parafunctional habits, such as bruxism, minimizes the risk of coronal restoration failure [19]. This study's selection criteria closely resembled those established by Alassar et al. 2022.

In this study, the High Impact Polymer Composite block CAD/CAM was used due to the weaker tooth anatomy of FPMs in early adolescence, characterized by wider canals and thinner dentin walls [23]. This material offered advantages such as resilience and the ability to be repaired intraorally [22]. This approach agreed with the findings of Zheng et al. 2021, who suggested that composite can be a viable alternative to ceramic materials due to its resilience similar to dentine and its ability to distribute stress more uniformly, and offers good fracture resistance [31].

Composite CAD/CAM endocrowns offered advantages such as conservative tooth preparation and preservation of periodontal health due to the high accuracy of marginal fit and supragingival marginal placement [32]. In contrast, both SSCs and PZCs required extensive tooth preparation [17]. Specifically, the reduction required for PZCs is greater than that for SSCs because PZCs necessitate a completely passive fit [17]. Also, subgingival preparation is required for both SSCs and PZCs, which may negatively impact the periodontium [16, 17].

Retention is a critical measure of restoration success. The present study investigated the retention of composite CAD/CAM endocrowns in children. While the follow-up period was one year, the author observed only one case of partial fracture with the debonding at six months. This outcome was similar to a case series conducted by Tzimas et al. 2018, who reported a partial fracture of a hybrid resin composite-ceramic endocrown after five months [25]. This fracture may be due to the lower fracture resistance of resin composite materials compared to other ceramic materials. This is consistent with a study reported by Altier et al. 2018, in which composite endocrowns exhibited less fracture resistance than ceramic materials [33]. However, composite CAD/CAM endocrowns demonstrated a more desirable failure mode than ceramic endocrowns.

In contrast, Mumoza-Sanchez et al. 2020 observed that CAD/CAM endocrowns made of either IPS e.max CAD or Vita Enamic showed no failures in the coronal restorations after 12 months of follow-up [34]. Similarly, Alassar et al. 2022 found that after 2 years of follow-up, all IPS e.max CAD endocrowns were still retentive and had no fracture for the duration of the study. However, the use of ceramic materials often requires greater tooth reduction [19]. Therefore, composite endocrowns offered a potential balance between preserving tooth structure and achieving acceptable longevity, particularly during early adolescence.

The findings of this study disagreed with Alassar et al. 2022, who reported a higher failure rate in the control group (2 out of 5 restorations) at the 12-month follow-up with direct composite restoration of endodontically treated FPMs in children of similar age groups [19]. While both materials were composites, Indirect composite endocrowns offered greater coverage and retention, and less microleakage compared to direct composite restorations, as supported by Azeem and Sureshbabu. 2018 [35].

The data from this research corroborated the conclusions by Talekar et al. 2023 who reported that at the six-month follow-up, two SSCs were lost, while at the 12-month follow-up, two PZCs were lost. The study attributed the cause of crown loss to adhesive failure [18]. Another study by Geduk et al. 2023 reported that during the 18-month follow-up, one PZC was lost at the 13-month follow-up due to decementation [17]. Additionally, a retrospective study by Discepolo and Sultan. 2017 evaluated 155 SSCs, reporting a 12% failure rate (primarily debonding) over 6–99 months of follow-up [16].

As per other treatment options proposed for the treatment of mutilated FPMs, one option was the post-core crown. The insertion of a post and core involves enlarging the canal system. Sometimes, these canals are curved and have different angles [20]. This type of crown can lead to significant tooth reduction, potential root perforation, and an increased risk of root fracture [36]. This makes the post-core crown less ideal for FPMs in early adolescent patients.

Postoperative pain is an essential indicator of patient comfort and treatment success. The postoperative pain parameter was measured and showed a significant decrease after one week of RCT but remained stable thereafter. Unexpectedly, other studies examining different types of crowns placed in FPMs (such as Geduk et al., 2023; Heidari et al., 2019; Alassar et al., 2022; Talekar et al., 2023; Discepolo and Sultan, 2017; and Koleventi et al., 2018) did not measure postoperative pain during the follow-up periods [16–19, 37, 38]. However, pain is a negative experience, and minimizing it is crucial for patient comfort and satisfaction. Moreover, it evaluated the quality and effectiveness of both RCT and various types of crowns used in FPMs.

In this study, composite CAD/CAM endocrowns achieved higher satisfaction scores than stainless steel crowns (SSCs), primarily due to superior aesthetics. A customized 4-point scale was used to evaluate satisfaction, focusing on objective criteria such as pain, chewing capability, and aesthetics. Parents and patients prioritized pain relief and improved mastication over aesthetics when reporting satisfaction reasons. Notably, some children preferred SSCs, citing perceived strength, highlighting a divergence in preferences.

The findings of this research confirmed the results of a study conducted by Talekar et al. 2023 who assessed parental acceptance of the SSCs and PZCs only at 12 months [18]. The findings revealed that parents expressed greater satisfaction with PZCs compared to SSCs. The attractive appearance of PZCs and the assurance of not needing to replace the crown again were cited as the main reasons for parents favoring PZCs over SSCs. Conversely, the cost-effectiveness and quick application of SSCs were highlighted as positive factors influencing parental acceptance of SSCs.

A radiographic evaluation revealed no root fractures in either group. One composite CAD/CAM endocrown failed, resulting in periapical pathology, but was successfully retreated. One SSC was removed due to patient-reported pain despite radiographic normalcy, likely due to excess cement. Retreatment was offered, but the mother refused further follow-up.

This finding was comparable to Alassar et al. 2022, who observed no root fractures in either the IPS e.max CAD endocrown group or the direct composite group. However, unlike the endocrown group, two cases in the direct composite group exhibited RCT failure, likely due to composite shrinkage, which may be attributed to the more accurate marginal seal and the absence of shrinkage in the endocrown [19].

This finding was consistent with retrospective studies by Discepolo and Sultan. 2017 and Oh et al. 2020, who reported no root fractures in SSCs during follow-up periods of 6–99 months and 12–180 months, respectively. However, periapical pathology was observed in two studies associated with RCT failure [16, 39]. Additionally, a study by Sigal et al. 2020, who observed SSCs radiographically in a specific population (SHCN) and reported no root fractures [40].

The result of this study was partially supported by Geduk et al. 2023, who found no root fractures in teeth restored with PZCs and SSCs over 18 months [17]. However, unlike our findings, they observed minor discrepancies in crown margins, such as slight overhangs or undercuts, in a small number of crowns.

A limitation of this study is the brief follow-up duration, which restricts the evaluation of long-term outcomes. Thus, future research with longer follow-up periods is necessary. This study also did not evaluate the cost-effectiveness of composite CAD/CAM endocrowns compared to SSCs, an important factor for clinical decision-making. Additionally, the limited sample size might restrict the generalizability of these findings; therefore, larger studies are necessary to explore the effectiveness of composite CAD/CAM endocrowns.

## Conclusion

In this study, composite CAD/CAM endocrowns and SSCs demonstrated comparable clinical and radiographic outcomes for restoring endodontically treated FPMs in children, with no statistically significant differences observed in retention, radiographic root fracture, or postoperative pain, except for patient satisfaction. However, clinicians may consider composite CAD/CAM endocrowns when patient satisfaction, particularly concerning esthetics, is a primary treatment objective.

### Recommendation

- A larger sample size study is needed to increase the statistical power and generalizability of the findings regarding the effectiveness of composite CAD/CAM endocrowns in FPMs.
- Investigation of the clinical and radiographic performance of composite block CAD/CAM endocrowns and ceramic endocrowns for restoring FPMs to provide a more comprehensive comparison and to better assess the relative advantages of each material in pediatric endodontic restorations.

## Data Availability

All pertinent data are incorporated in this manuscript or the supplementary files. Dr. Basheer Ali (Basheer. ali@dentistry. cu.edu.eg) is the corresponding author and can be contacted for any additional data requests deemed reasonable.

## Abbreviations

FPMs: First Permanent Molars
SSCs: Stainless Steel Crowns
PZCs: Preformed Zirconia Crowns
SHCN: Special health care needs population
MIH: Molar Incisor Hypomineralization
CAD/CAM: Computer-Aided Design/Computer-Aided Manufacturing
RCT: Root Canal Treatment
CBCT: Cone Beam Computed Tomography
ICC: Intraclass Correlation Coefficient
